# *Ubx* Regulates Differential Enlargement and Diversification of Insect Hind Legs

**DOI:** 10.1371/journal.pone.0000866

**Published:** 2007-09-12

**Authors:** Najmus Mahfooz, Nataliya Turchyn, Michelle Mihajlovic, Steven Hrycaj, Aleksandar Popadić

**Affiliations:** 1 Department of Biological Sciences, Wayne State University, Detroit, Michigan, United States of America; University of Queensland, Australia

## Abstract

Differential enlargement of hind (T3) legs represents one of the hallmarks of insect evolution. However, the actual mechanism(s) responsible are yet to be determined. To address this issue, we have now studied the molecular basis of T3 leg enlargement in *Oncopeltus fasciatus* (milkweed bug) and *Acheta domesticus* (house cricket). In *Oncopeltus,* the T3 tibia displays a moderate increase in size, whereas in *Acheta,* the T3 femur, tibia, and tarsus are all greatly enlarged. Here, we show that the hox gene *Ultrabithorax* (*Ubx*) is expressed in the enlarged segments of hind legs. Furthermore, we demonstrate that depletion of *Ubx* during embryogenesis has a primary effect in T3 legs and causes shortening of leg segments that are enlarged in a wild type. This result shows that *Ubx* is regulating the differential growth and enlargement of T3 legs in both *Oncopeltus* and *Acheta*. The emerging view suggests that *Ubx* was co-opted for a novel role in regulating leg growth and that the transcriptional modification of its expression may be a universal mechanism for the evolutionary diversification of insect hind legs.

## Introduction

**Figure 1:**
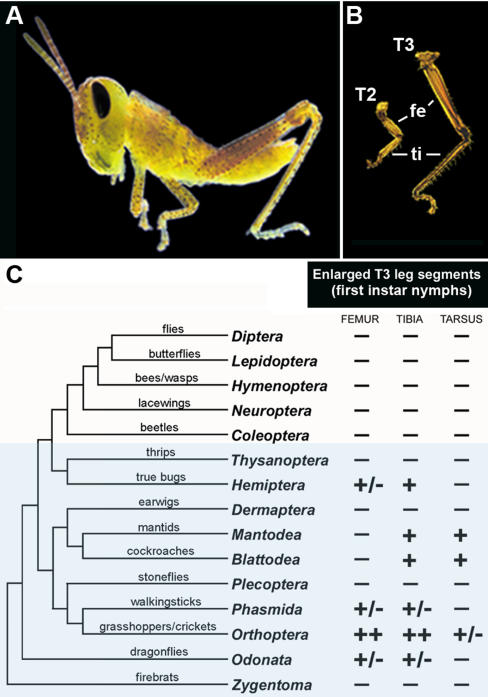
A trend toward increased size of insect hind legs is characterized by variation in both the identity and magnitude of enlarged segments. (A) First instar nymph of grasshopper *Schistocerca americana*. (B) Dissected nymphal T2 and T3 legs, showing enlarged femoral (fe) and tibial (ti) segments in the latter. (C) An abbreviated insect phylogeny illustrating a trend toward differential enlargement of hind leg segments. Plus and minus refer to the presence or the absence of increase in the T3 segment size (compared to forelegs), respectively. Two pluses indicate a great enlargement, while a +/− denotes a presence of size increase in some but not all of the members of the group. Note that the depicted leg size differential refers to that observed in first instar nymphs (for hemimetabolous groups) and first instar larvae (for holometabolous groups), and not to adult morphologies.

At present, we are only beginning to understand how developmental variation governs phenotypic diversity in nature [1]–[4]. To address this fundamental question, we focused on insect hind (T3) legs as a model for studying morphological evolution. Together with the other two leg pairs (T1 and T2), insect hind legs exhibit a modular organization and are composed of five segments (coxa, trochanter, femur, tibia, and tarsus, followed by claws). While the number and arrangement of these segments are highly conserved, their relative sizes and functional anatomies are very diverse, reflecting different adaptive responses. Among the three pairs, the hind legs exhibit an extraordinary range of morphological diversity, encompassing both the small-scale (cuticle coloration, bristle pattern) and large-scale (overall change in size and shape) morphological differences. The most noticeable aspect of their evolution is the *differential enlargement* of hind leg segments compared to their T1 and T2 counterparts [5]. A prime example of this evolutionary trend is found in orthopterans (grasshoppers and crickets), an insect order easily recognized by the presence of greatly enlarged “jumping” T3 legs (Fig. 1A).

The differential growth is often first observed during embryonic development resulting in first instar nymphs and larvae that have larger hind legs [5]. In many species allometric growth continues during post-embryonic development, so much so, that the majority of adult insects have T3 legs that are at least slightly enlarged compared to forelegs [6], [7]. Our current analysis is focused on a condition in first instar nymphs that allows us to infer the direct effect of embryonic gene expression patterns on leg morphology. The observed large variation in leg size is accomplished by varying both the number and magnitude of enlarged segments. Depending on the species, the increase in size can encompass three, two, or just one leg segment (Fig. 1C). For example, in grasshopper, the overall size increase is accomplished by differential enlargement of only two leg segments, the femur and tibia (Fig. 1B), while the rest of T3 segments are similar in size to their foreleg counterparts. The magnitude of the enlargement can range from a 200% to 5% increase of the size of corresponding T1/T2 segments. Furthermore, where the majority of hemimetabolous groups exhibit this differential increase, there are also some lineages evolving in the opposite direction with hind legs becoming more similar to forelegs (Dermaptera, earwigs, and Thysanoptera, thrips; Fig. 1C). These extraordinary differences in the size of T3 legs provide an excellent system to study the molecular origins of phenotypic change in nature. The question remains, what is the actual molecular mechanism that governs the divergence of insect hind legs and is it universal or species-specific?

Recently we discovered a strong correlation between differentially enlarged T3 leg segments and differential embryonic expression of two homeotic genes, *Ultrabithorax* (*Ubx*) and *abdominal-A* (*abd-A*) [5]. These observations led us to propose that spatial and temporal changes in the expression of one or both of these genes may have been instrumental in the evolution of insect hind legs. To test this hypothesis, we have now studied the molecular basis of T3 leg enlargement in *Oncopeltus fasciatus* (milkweed bug) and *Acheta domesticus* (house cricket). Both species undergo hemimetabolous mode of development, with hatched first instar nymphs looking like miniature adults. Here, we use a functional test (RNA interference, RNAi) to show that *Ubx* is responsible for the differential growth and enlargement of T3 legs in both *Oncopeltus* and *Acheta*. More specifically, changes in *where* and *when Ubx* is expressed can account for which segments(s) will increase in size as well as the magnitude of their increase. This finding indicates that transcriptional modification of *Ubx* expression may be a common mechanism for the evolutionary diversification of insect hind legs.

## Materials and Methods

### Cloning and Sequence Analysis of cDNA Fragments

Mixed stage embryos of *O. fasciatus* (milkweed bug) and *A. domescticus* (house cricket) were used for total RNA extraction. The production of cDNA, RT-PCR, and cloning were performed according to Li and Popadic [8]. Two degenerate primers targeting the highly conserved amino acid motifs (that includes the homeodomain region), FYPWM (5′ AYC ACA CRT TYT AYC CCT GGA 3′) and WFQNRR (5′ GCT CTA GAC GIC GRT TTT GRA ACC A 3′) were used to amplify *Ubx* and *abd-A* fragments. Twenty copies of each clone obtained were recovered and sequenced. All obtained nucleotide sequences were compared with each other and to previously described *Ubx* and *abd-A* homologs in GeneBank using MacVector 6.0.1 (Kodak) software. No evidence of paralogous copies for either gene was found.

FirstChoice RLM-RACE kit (Ambion) was used to obtain longer cDNA fragments, which also include the 3′end of each gene,. 3′ RACE PCR reactions were carried out using gene specific primers and anchor primers supplied with the kit. The cloning and sequence analysis of each clone was accomplished as described above.

### *In situ* Hybridization

For both gene fragments (*Ubx* and *abd-A*), an antisense RNA probe was generated using Riboprobe Combination System-T3/T7 kit (Promega) with digoxigenin-UTP (Roche, Mannheim, Germany). The probe was purified using a Mini Quick RNA column (Roche). The *in situ* hybridization procedure was performed as described in Li and Popadic [8]. Hybridization with the probe was done at 55°C (*Oncopeltus*) and 58°C (*Acheta*) for 24 h followed by an overnight incubation in hybridization buffer alone to reduce background. To visualize the RNA pattern generated, anti-digoxigenin antibody conjugated with alkaline phosphatase was used (Roche) with 2-hour incubation at room temperature. The signal was revealed by NBT+BCIP color reaction (Roche). Stained embryos were then cleaned from residual yolk as much as possible and mounted on a microscope slide in a drop of Aqua Polymount (Polysciences Inc). We examined several hundred stained embryos at various stages to infer the general expression pattern.

### RNA interference

Maternal RNA interference (RNAi) was performed by injecting double-stranded RNA (dsRNA) into females as described by Liu and Kaufman [9] and Angelini et al. [10]. The RNAi procedure was optimized using a previously cloned fragment of the *Oncopeltus Scr* gene (kindly supplied to us by C. Hughes and T. Kaufman). The generated *Scr*-depleted phenotypes were identical to ones reported by Hughes and Kaufman (2000), who used an embryonic RNAi approach [11]. To analyze the function of *Ubx* and *abd-A*, the dsRNA of two different lengths was synthesized for each gene. The longer transcript (310–330 bp) encompassed the homeodomain and the carboxyl terminus of the gene, while the shorter transcript contained mainly the 3′ untranslated gene region (120–130 bp). Both types of transcript produced essentially the same RNAi phenotypes. To control for the possible side effects of the RNAi on leg growth, we injected 375 bp dsRNA of the jellyfish GFP gene. The morphology and leg size of these injected embryos was indistinguishable from that of the wild type.

We examined a total of 581 (for *Ubx*-RNAi) and 982 (for *abd-A*-RNAi) hatched first instar nymphs in *Oncopeltus*, and a total of 586 (for *Ubx*-RNAi) embryos in *Acheta*. For each gene, we established the distribution and proportion of depleted phenotypes per clutch. A portion of embryos from clutches 3–5 (which is when RNAi phenotypes appear at 100%) were set aside and examined for the presence of *Ubx* and *abd-A*, respectively. In each instance, a complete absence of the signal was observed. Hence, the observed phenotypes in clutches 3 and on can be attributed to a complete absence of a respective gene expression.

### Leg measurements

To examine the effect of *Ubx* depletion on leg size, T2 and T3 legs from twenty first instar nymphs (*Oncopeltus*) or late embryos (*Acheta*) were dissected and measured. The data were analyzed by ANOVA (SPSS Version 11.5). Duplicate measurements were made for each embryo or nymph. The second measurements were made after all of the embryos had been previously measured, and the order of the embryos was randomized. Data were first analyzed for repeat measurements using ANOVA. As there was no significant difference between the first and second, we pooled the data for the final ANOVA analysis. Means and standard errors were calculated as ∑ length/n and S.D./√n, where n is the number of embryos analyzed. The effect of *Ubx* depletion on leg segment size was evaluated using one-tail T-test.

## Results

### Divergent embryonic expression of *Ubx* in *Oncopeltus*

**Figure 2:**
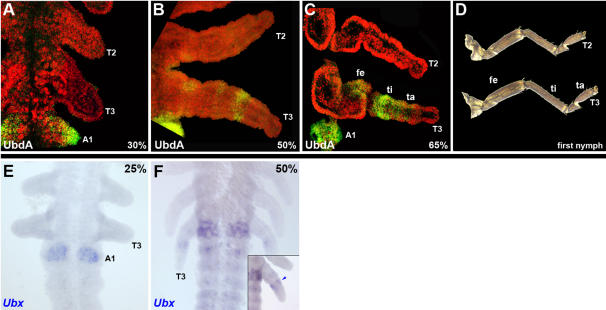
Expression of *Ubx* in *Oncopeltus.* (A–C) Combined *UbdA* (*Ubx*+*abd-A*) expression (green) visualized by FP6.87 antibody. (D) Dissected T2 and T3 nymphal legs. (E–F) *Ubx* mRNA accumulation pattern at 25% and 50% development, respectively. Whereas early expression is localized solely in the A1 segment (E), the later stages also exhibit signal in T3 legs and posterior abdomen (F).

We first analyzed the combined expression patterns of *Ubx* and *abd-A* (collectively referred to as *UbdA*) by using a cross-reactive antibody that detects both proteins [12]. During the first 30% of development, the *UbdA* signal is observed solely in the abdomen, with the anterior boundary in the first abdominal segment, A1 (Fig. 2A). This abdominal expression continues throughout embryogenesis. From 30–50% of development, *UbdA* begins to be additionally expressed in the hind (T3) legs (Fig. 2B). More specifically, the primary protein accumulation is observed as a narrow ring in mid-distal leg region. In addition, there are also two proximal patches of *UbdA* expressing cells. During later stages of development, as leg segmentation becomes complete, the hind leg pattern is again modified. The strongest expression is now observed in the tibial segment, accompanied by much lower levels in portions of femur and tarsus (Fig. 2C). This dynamic embryonic pattern correlates well with the morphology of T3 legs in first instar nymphs: the primary enlarged segment is the tibia (20% increase), whereas the femur and tarsus are only slightly longer compared to forelegs (Fig. 2D). Thus, in *Oncopeltus* there is a tight association between the observed differential hind leg expression of *UbdA* and the differential enlargement of these appendages in first instar nymphs.

In order to distinguish between the *Ubx* and *abd-A* individual patterns, we cloned partial cDNA fragments from each and used them to study their respective mRNA expression. Angelini et al. recently reported that *abd-A* expression is restricted to the abdomen only, extending from posterior A1 to A10 segment [10]. In contrast, *Ubx* is localized solely in the A1 segment at the germ band stage (Fig. 2E). However, as appendages begin to elongate, differential *Ubx* expression can be observed in the T3 legs (inset, Fig. 2F). There is also a very low level of signal in clusters of *Ubx*-expressing cells in segments A2–A8 (Fig. 2F; [10]). These results show that the previously described combined *UbdA* pattern is actually generated by a distinct *Ubx* expression in T3 legs and A1 segment and by separate *abd-A* expression from posterior A1 to A10 segment. Furthermore, the temporal order of *Ubx* expression suggests that this gene may have two very different functions in milkweed bugs: an early one in the abdomen, and a later role in hind legs.

### *Oncopeltus Ubx* has two distinct roles: one in the A1 segment and another in T3 legs

**Figure 3:**
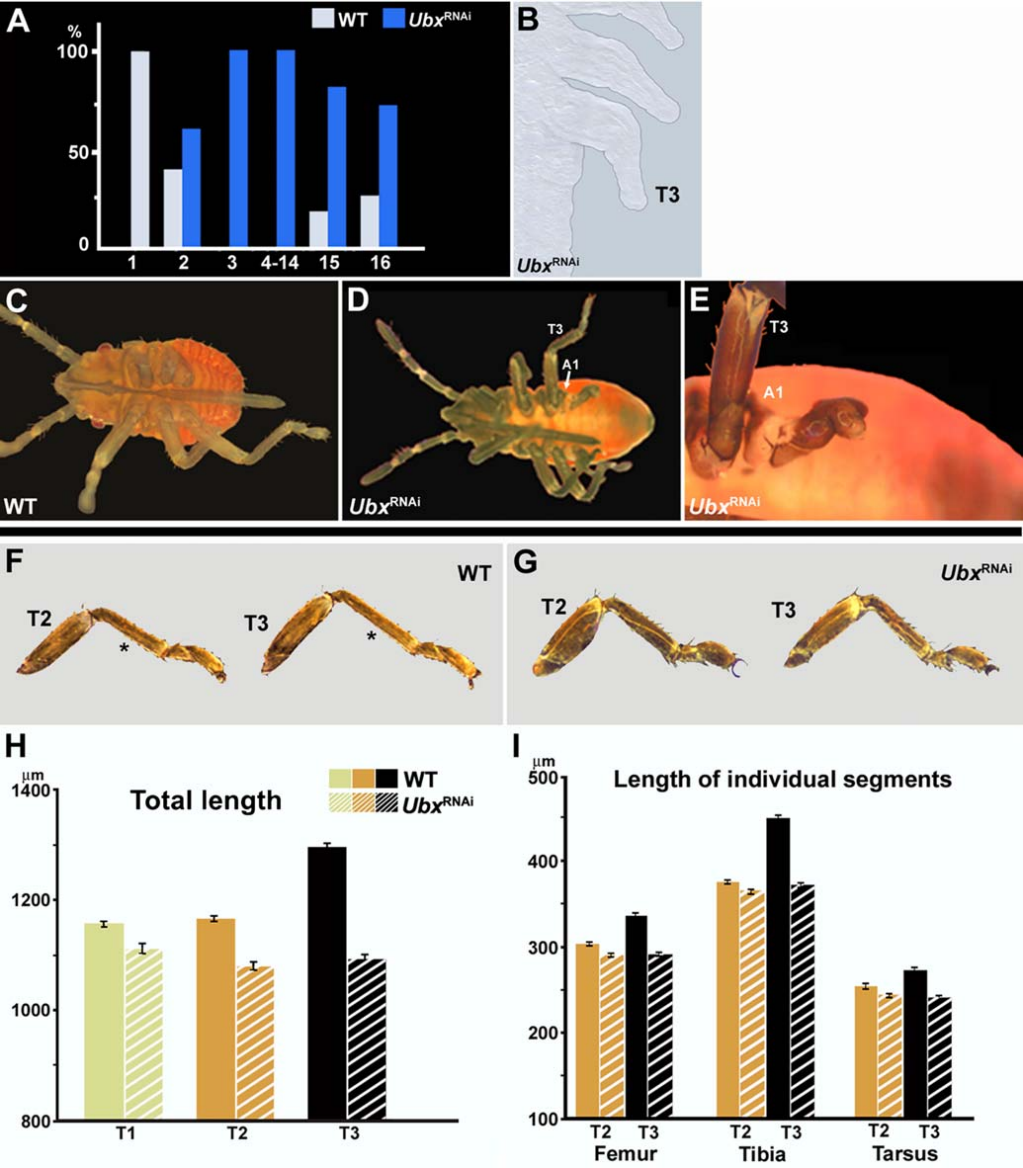
Phenotypic effects *of Ubx-*RNAi in *Oncopeltus.* (A) Distribution and proportion of embryos with *Ubx* depleted phenotypes per clutch, starting with the first clutch after injection. (B) There is a complete absence of embryonic *Ubx* expression in the depleted embryos from the fourth clutch on. (C–D) Morphology of first instar nymphs in wild type and *Ubx*-depleted individuals. (E) The magnified image corresponding to panel D, showing the presence of an ectopic appendage on the A1 segment. (F–G) Dissected T2 and T3 legs of wild type and *Ubx*-RNAi first nymphs, respectively. The star indicates the tibial segment. In *Ubx*-RNAi nymphs, there is a visible size decrease in the hind legs, making these appendages very similar to T2 legs. (H) The differences in the total leg lengths in wild type and *Ubx*-RNAi hatchlings. (I) The differences in the size of individual T2 and T3 leg segments between wild type and *Ubx*-RNAi nymphs. In the latter, the overall length decrease in the hind legs is primarily caused by the shortening of the tibia, and to a much lesser degree the femur and tarsus. In both panels (H and I), the differences in leg length between wild type and RNAi first instar nymphs are statistically significant.

To examine the functional significance of the observed *Ubx* expression patterns further, we have performed maternal RNA interference (RNAi) experiments [9]. By using this approach, it is possible to effectively block the expression of the targeted gene in the developing *Oncopeltus* embryos. We have first analyzed the efficiency and penetrance of *Ubx*-RNAi (Fig. 3A). Following the injections, *Ubx*-depleted embryos start appearing in the second clutch. Note that by the third clutch the RNAi reaches a complete penetrance, and from clutches 3–14 all of the embryos that hatch out exhibit a mutant phenotype (Fig. 3A). To confirm that the observed changes are indeed caused by RNAi, we tested embryos from clutches 4–8 for *Ubx* expression. These embryos display a complete absence of *Ubx* (Fig. 3B), thus providing a strong evidence that the observed *Ubx*-depleted morphologies are comparable to knock-out phenotypes.

There are two key features that characterize *Ubx*-RNAi first instar nymphs (Fig. 3D), one in abdomen and one in T3 legs. First, there is now the presence of an additional leg-like appendage on the A1 segment (Fig. 3E in this report, [10]). In contrast, the wild type morphology is characterized by a completely limbless abdomen (compare Figs. 3C and 3E). The limbless morphology is generated by repressing *Distal-less*, *Dll*. In *Ubx* depleted embryos, the *Dll* repression is released, which in turn leads to formation of ectopic leg-like appendages (Fig. S1, [10]). In addition, the first abdominal segment also adopts dorsal pigmentation resembling the T3 segment pattern [10]. All of these observed changes suggest that *Ubx*-depleted embryos display a transformation of A1 toward a thoracic (presumably T3) identity ([10]; this report). Conversely, these results show that the early role of *Ubx* in *Oncopeltus* is to convey the abdominal identity to A1 segment, similar to previous findings in *Drosophila* [13], *Tribolium* [14], [15], and *Manduca* [16].

The second major feature of the *Ubx*-RNAi nymphs is the altered morphology of the hind legs. *Oncopeltus* T3 legs are characterized by a moderate degree of enlargement, primarily due to the differential increase in the length of the tibia (Fig. 3F). In comparison, in *Ubx*-RNAi treated nymphs the hind legs are now visibly smaller and very similar to T2 legs (Fig. 3G). With regard to total length (Fig. 3H), the hind legs exhibit the largest length decrease (15.7%)–consistent with the differential expression of *Ubx* in these appendages. There is also a small reduction in forelegs (∼4% and 6.2%, respectively). Because *Ubx* is never expressed in T1/T2 legs, the decrease in their lengths cannot be attributed directly to the effects of its depletion. Stern and Emlin [17] suggested a model in which autonomous organ growth is regulated by feedback from other growing organs. Our observation of the reduction in length of forelegs in *Ubx*-RNAi nymphs is consistent with such a model. In addition, it is possible that the growth of ectopic legs on A1 segment affects the growth of thoracic appendages.

In order to further characterize the role of *Ubx* in hind legs, we examined the length of individual leg segments in wild type and *Ubx*-depleted first instar nymphs (Fig. 3I). The primarily enlarged segment in *Oncopeltus* is the T3 tibia (20% increase), whereas the femur and tarsus are only slightly longer compared to forelegs (11% and 7.5% increase, respectively). *Ubx* expression in the hind legs closely matches this pattern of enlargement–while strong *Ubx* signal is observed in the whole tibia, it is present only in a few clusters of cells in the femur and tarsus (Fig. 2). Hence, the *Ubx* depletion should have the largest effect on the T3 tibia, and a much lesser effect on T3 femur and tibia. As shown in Fig. 3I, the most affected segment is indeed the T3 tibia, followed to a much lesser degree by the T3 femur and tarsus. Note that the corresponding T2 leg segments are only marginally affected. For example, in wild type, the T3 tibia is significantly longer (20%; p<<0.01), than the T2 tibia (450±5.4 um and 373±6.7 um; respectively). In *Ubx*-RNAi first instar nymphs, however, there is a large decrease in the length of the T3 tibia (from 450±5.4 um to 365±2.2 µm), whereas the T2 tibia is only slightly affected (from 373±6.7 um to 361±3.3 µm). As a result, the *Ubx*-depleted T3 and T2 tibial segments are not significantly different (Fig. 3I). In a similar fashion, the T3 femur and tarsus in *Ubx*-RNAi nymphs exhibit a distinct and noticeable shortening. These findings provide direct evidence that *Ubx* is regulating the differential growth of hind legs in *Oncopeltus*.

As shown in Fig. 2, the pattern of expression suggests that *Ubx* has two distinct embryonic roles in milkweed bugs, one in the abdomen and the other in thorax. The present RNAi studies confirm this prediction. First, *Ubx* controls the identity of A1 segment. Consistent with that role, the A1 segment in *Ubx*-RNA nymphs is transformed into T3 segment ([10]; this study). Second, we document a novel function in the thorax, where *Ubx* controls the size of T3 legs. Note that the identity of the whole T3 segment is not being affected. This is consistent with the observation that *Ubx* is localized to distinct regions of hind legs only.

### *Ubx* also regulates the size of orthopteran “jumping” legs

**Figure 4:**
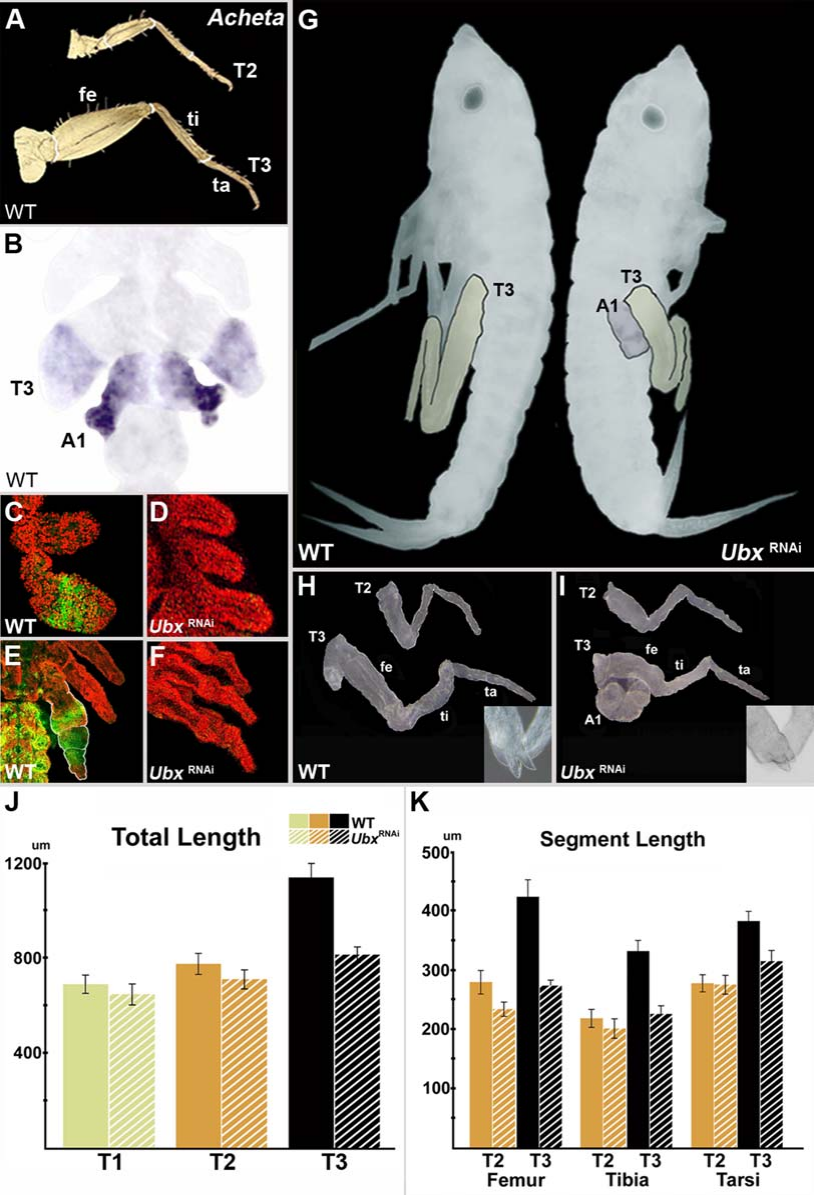
Phenotypic effects *of Ubx* RNAi in *Acheta.* (A) Dissected T2 and T3 legs of first instar nymphs showing differential enlargement of femoral (fe), tibial (ti), and tarsal (ta) segments. (B) Embryonic *Ubx* mRNA expression is localized in the T3 leg and A1 segment. (C–F) Compared to wild type, there is a complete absence of *UbdA* signal in both early (C–D) and late (E–F) *Ubx*-depleted embryos, indicating a complete silencing of expression throughout embryogenesis. (G) Wild type and *Ubx*-RNAi embryos at 85% development, displaying clearly reduced hind legs as well as ectopic appendages on A1. To better visualize their size, the T3 legs and A1 appendage were artificially colored. (H–I) Dissected T2 and T3 legs in wild type (H), and *Ubx* embryos (I). Insets show that the paired spurs at the tibia-tarsus joint remain present in the depleted embryos. (J–K) The differences in the total leg lengths and in the lengths of individual segments between wild type and *Ubx*-RNAi hatchlings, respectively. (J) The T3 leg exhibits the most significant overall overall shortening. (K) This decrease in the total length is primarily caused by the shortening of the femur and tibia, and to lesser degree of the tarsus.

Orthopterans exhibit the largest increase in T3 leg size among insects (Fig. 1a) and represent a natural choice for further investigation of *Ubx* function. The house cricket *Acheta domesticus* displays a typical orthopteran “jumping” leg morphology in which the femoral, tibial, and tarsal hind leg segments are greatly enlarged compared to corresponding forelegs counterparts (Fig. 4A). As we recently reported [5], the T3 leg in *Acheta* also exhibits differential *UbdA* expression. In order to distinguish between the individual gene patterns, we have now analyzed embryonic *Ubx* patterning in this species. The observed signal is localized mainly in T3 legs and A1 segments (Fig. 4B). This pattern suggests two distinct roles, one in abdomen and one in thorax, analogous to findings in *Oncopeltus*. In hind legs, expression starts at the limb bud stage (Fig. 4C). By the time leg segmentation is complete, *Ubx* becomes localized in the femur, tibia, and proximal tarsus (Fig. 4E), the segments that are differentially enlarged in first instar nymphs [5], [18]. To examine the functional significance of *Ubx* expression in *Acheta*, we utilized the previously described RNAi analysis. As indicated by the absence of signal in T3 legs from early to late embryogenesis (Figs. 4D and 4F), the RNAi application suppresses *Ubx* expression throughout cricket development. *Ubx* embryos do not hatch out, similar to situation in *Tribolium* [19], however they do complete about 85% of development and their phenotype can be scored.

There are two key morphological changes that characterize *Ubx*-depleted embryos (Fig. 4G). First, an ectopic leg develops on the A1 segment. This result is consistent with the observations in *Oncopeltus* and suggests that the early role of *Ubx* is to regulate abdominal identity in A1 by repressing leg development in this segment. Second, hind legs show a reduction in size throughout embryogenesis. The T3 legs are now visibly smaller and appear to be very similar to forelegs (Fig. 4G). There is a pronounced difference in the length of the femur and tibia, and to a lesser degree, of the tarsus between wild type and *Ubx*-depleted hind legs (compare Fig. 4H vs. Fig. 4I). Note that in addition to their size differential, wild type hind legs can also be differentiated from their foreleg counterparts by the presence of two spurs on the tibia, close to the joint between the tibia and tarsus (Fig. 4H, inset). The paired spurs are still present in *Ubx*-depleted T3 legs (Fig. 4I, inset), although these appendages are now visibly reduced. This result suggests that while the size of hind legs is affected in *Ubx*-RNAi embryos, their identity is not.

In crickets, the “jumping” T3 legs are by far the longest appendages averaging 1136.7 µm (Fig. 4J, solid bars). Compared to T2 legs, they are 47% longer. These legs also exhibit the most pronounced size reduction in *Ubx* depleted embryos (Fig. 4J, stripped bars). The average length of *Ubx-*RNAi T3 legs is 773.5 µm representing a 28.5% reduction. The shortening of the forelegs is much smaller, ranging from 8.4% in T2 to 6.2% in T1 legs. As a consequence, the overall enlargement of hind legs drops from 47% to 14.6%. These results further corroborate the role of *Ubx* in the differential growth of T3 legs. Similar to the observations in *Oncopeltus*, there is also a small reduction in the forelegs.

The overall decrease in length of T3 legs results from the shortening of three individual leg segments, namely the femur, tibia, and tarsus (Fig. 4G). Compared to wild type, these segments exhibit a reduction in length that ranges from 35.5% (femur) to 32.1% (tibia) to 17.7% (tarsus). This difference in the degree of shortening of hind legs correlates well with *Ubx* expression: whereas *Ubx* is expressed throughout the femur and tibia, in the tarsus it is localized in the proximal region only [5]. Accordingly, the former two segments should exhibit a more pronounced size reduction than the latter.

The present study reveals that *Ubx* has two distinct embryonic roles in *Acheta*. First, *Ubx* controls the identity of A1 by suppressing limb development on this segment. This result is consistent with the early expression pattern and corroborates the findings in *Oncopeltus*. Second, we confirm a novel function in regulating the differential growth of hind legs that was predicted on the basis of *Ubx* expression patterns in house crickets [5]. Previous studies in a grasshopper *Schistocerca gregaria* and cricket *Gryllus bimaculatus* also noted the association between *Ubx* expression and its localization in enlarged segments of T3 legs [12], [20]. This combined insight from the present functional study and the earlier expression analyses strongly suggests that the role of *Ubx* in the enlargement of hind legs is not restricted to crickets, but is common to the whole orthopteran lineage.

## Discussion

### The role of *Ubx* in diversification of insect hind legs

In the hemimetabolous mode of development, the key features of the adult body plan are largely established during embryogenesis. These species also encompass some of the most morphologically diverse insect lineages, making them suitable to study how developmental variation influences phenotypic variation. One of the key trends in insect evolution is the differential enlargement of their hind legs, which exhibit a wide range of taxon- and species-specific differences. These differences are established during embryogenesis [5], [7], when hatched first instar nymphs already display variation in which segments become enlarged as well as in the magnitude of their size increase (Fig. 1C). Studies in the past decade have also revealed a strong association between embryonic *Ubx* expression and differentially enlarged T3 leg segments in several hemimetabolous species [5], [12]. On the basis of these observations, it was proposed that *Ubx* may have acquired a novel role in the evolution of hind legs [5]. To test this hypothesis, we examined embryonic *Ubx* function in two divergent insect lineages, hemipterans (*Oncopeltus*) and orthopterans (*Acheta*). Our results show that in addition to its previously described role in the abdomen [10], [21], *Ubx* also regulates the differential growth of T3 legs in both *Oncopeltus* and *Acheta*. This finding, combined with the previous expression and functional studies in *Drosophila* and *Tribolium* [14]–[16], [22], [23], indicates that the role of *Ubx* in T3 legs may be common to both the hemimetabolous and holometabolous species. In other arthropods *Ubx* is associated with the establishment of distinct segmental and appendage identities [24]–[29]–but not with the diversification of limb morphology. Thus, *Ubx* function in T3 legs may be an evolutionary novelty in Insecta, responsible for the large morphological variation in the size of these appendages that exists in nature.

**Figure 5:**
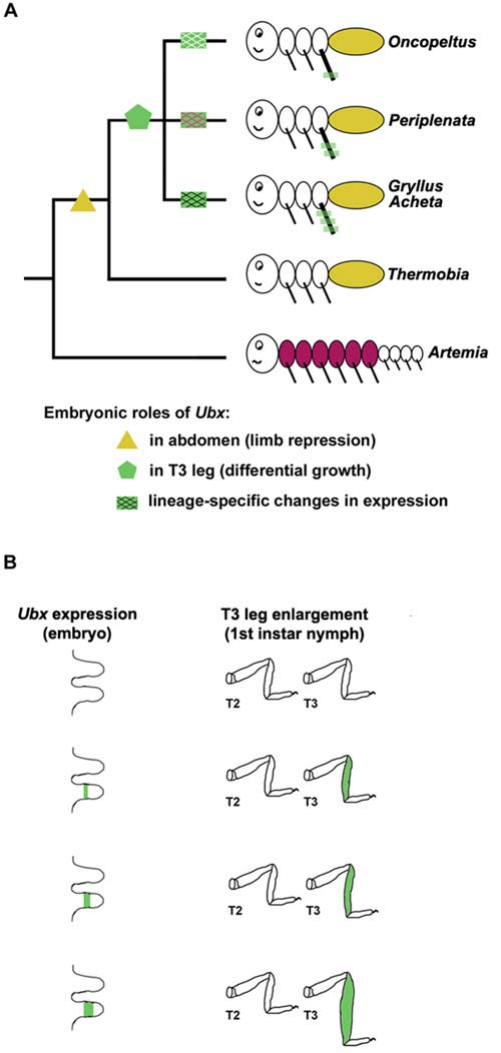
The embryonic roles of *Ubx* in the evolution of insect body plan. (A) During early insect evolution, *Ubx* gained two new functions: one in the abdomen (limb repression) and another in hind legs (differential growth). The striped rectangles depict lineage-specific modulations of *Ubx* expression in different T3 leg segments. This, in turn, provided positional information as to which regions of the hind legs will be enlarged. (B) A model of how *Ubx* may regulate the differential enlargement of hind legs. The degree of enlargement is directly proportional to the expansion of *Ubx* expression–the larger the expression, the bigger the affected segment(s).

Classical studies in *Drosophila* highlighted the role of Hox genes as major developmental pathway switches–leading to a traditional view of these transcriptional factors acting as selector genes [13], [21], [30]. More recent work has revealed that Hox genes may act at all levels in the developmental hierarchy, even down to a single cell [1], [3], [31]–[34]. Consequently, the current view suggests that their morphological effect can range from dramatic (if they act as selector genes) to rather subtle (if they act as cell-type switches). The embryonic roles of *Ubx* in *Oncopeltus* and *Acheta* illustrate as to how such a duality of action (both as a selector and a modifier) shaped the evolution of the insect bauplan. As shown in Fig. 5A, a change in its protein structure enabled *Ubx* to gain a new function in the suppression of limb development [35], [36]. This was one of the instrumental steps in the formation of the limbless abdomen and establishment of the tripartite insect body plan. Note that the extent of *Ubx* function in the abdomen is defined by its expression pattern. As illustrated in Fig. 2E, during early development *Ubx* is expressed throughout the A1 segment only. Consistent with this observation, the A1 segment is transformed toward a thoracic identity in *Ubx*-RNAi first instar nymphs (Fig. 3D–E)–showing that *Ubx* acts as a selector gene in this segment [10]. Hence, the selector role is a consequence of both the acquisition of a new function (limb repression) and the localization of expression in the A1 segment. Accompanying the diversification of insect lineages, *Ubx* gained a novel function in the development of hind legs. We propose that *Ubx* was first co-opted as a regulator of tissue growth (Fig. 5A). This was followed by wide, taxon-specific changes in its regulation. The evolution of expression domains encompassed one or more segments and led to the establishments of specific patterns in various insect lineages (Fig. 5A, stripped boxes). This, in turn, provided positional information as to which regions of hind legs will be enlarged. At the same time, evolution of *Ubx* regulation also included changes in the timing of its expression during embryonic development. For example, its expression in *Oncopeltus* starts rather late, when leg segments are already established, and is localized primarily in the T3 tibia. This pattern matches the morphology of first instar nymphs, which have a moderately enlarged tibia (Fig. 2B). In *Acheta* we observed different dynamics with expression starting early and then becoming localized in the T3 femur, tibia, and proximal tarsus–coinciding with segments that are greatly enlarged in first instar nymphs (Fig. 4A). In both species, *Ubx*-RNAi results in the shortening of hind legs that is consistent with the observed expression patterns–the primary reduction of the tibia in *Oncopeltus* and the femur, tibia, and tarsus in *Acheta*. These results show that a second role of *Ubx* during insect embryogenesis is to regulate the differential enlargement of T3 legs. Note that while the morphology of these appendages has been significantly altered–their identity has not. As illustrated in Fig. 4H–I (insets), *Ubx*-RNAi embryos still have paired spurs at the femur-tibia boundary, similar to wild types. Hence, in this instance, *Ubx* acts as a global modifier of leg morphology by regulating the size of T3 leg segments. A similar, but much more subtle role was observed in *Drosophila* where differences in the levels of *Ubx* expression are associated with a species-specific trichome pattern on the femur of T2 legs [37]. These results suggest that both gross (segment size) and fine (segment trichome pattern) features of insect leg morphology may evolve as a result of changes in the expression patterns of a hox gene.

The present finding of *Ubx* involvement in the development of insect hind legs has two significant implications for studies of morphological evolution. First, it demonstrates that alterations of hox gene expressions can actually occur with a surprising frequency in a wide range of species in nature. The observed changes in *Ubx* are very dynamic, including both the gain of new domains in some instances and loss of existing domains in others (Fig. 1C). Second, it illustrates how expansions of *Ubx* expression along the proximal-distal axis can lead to large-scale phenotypic differences in leg size. A model in Fig. 5B depicts this process, invoking a set of steps, each of which is characterized by a gradual expansion of the *Ubx* domain in embryonic T3 legs. As a consequence, there is a steady increase in the length of hind legs in first instar nymphs. Such a model also helps us to visualize and understand how small, population-level differences could, over time, lead to large morphological differences. Our data demonstrate that significant variation in leg size can result from alterations in the timing and duration of expression of a single gene. While additional modifying loci are likely involved in the divergence of insect hind legs, the emerging view suggests that evolution of even complex phenotypes may have a rather simple origin.

## Supporting Information

Figure S1 (A) Aligned sequences of cloned *Ubx* and abd-A cDNA fragments from *O. fasciatus* and *A. domestica*. Abbreviations: Dm, *Drosophila melanogaster*, Tc, *Tribolium castaneum*, Jc, *Junonia coenia*, Of, *Oncopeltus fasciatus*, Ad, *Acheta domesticus*, and Gb, *Gryllus bimaculatus*. (B–D) Distal-less (Dll) expression in *Oncopeltus* wild type, *Ubx* RNAi, and abd-A RNAi embryos, respectively. In depleted embryos, Dll expression is released in A1 (C), and A2–A8 (D) segments.
(4.16 MB TIF)

## References

[1] Carroll SB, Grenier JK, Weaherbee SD (2001). From DNA to diversity..

[2] Wilkins AS (2002). The evolution of developmental pathways: Sinauer Associates, Inc..

[3] Hughes CL, Kaufman TC (2002). Hox genes and the evolution of the arthropod body plan.. Evol Dev.

[4] Pearson jC, Lemons D, McGinnis W (2005). Modulating Hox gene functions during animal body patterning.. Nature Reviews Genetics.

[5] Mahfooz NS, Li H, Popadic A (2004). Differential expression patterns of the hox gene are associated with differential growth of insect hind legs.. Proc Natl Acad Sci U S A.

[6] Snodgrass RC (1952). A textbook of arthropod anatomy..

[7] Heming BS (2003). Insect development and evolution..

[8] Li H, Popadic A (2004). Analysis of nubbin expression patterns in insects.. Evol Dev.

[9] Liu PZ, Kaufman TC (2004). hunchback is required for suppression of abdominal identity, and for proper germband growth and segmentation in the intermediate germband insect Oncopeltus fasciatus.. Development.

[10] Angelini DR, Liu PZ, Hughes CL, Kaufman TC (2005). Hox gene function and interaction in the milkweed bug Oncopeltus fasciatus (Hemiptera).. Dev Biol.

[11] Hughes CL, Kaufman TC (2000). RNAi analysis of Deformed, proboscipedia and Sex combs reduced in the milkweed bug Oncopeltus fasciatus: novel roles for Hox genes in the hemipteran head.. Development.

[12] Kelsh R, Weinzierl RO, White RA, Akam M (1994). Homeotic gene expression in the locust Schistocerca: an antibody that detects conserved epitopes in Ultrabithorax and abdominal-A proteins.. Dev Genet.

[13] Lewis EB (1978). A gene complex controlling segmentation in *Drosophila*.. Nature.

[14] Bennett RL, Brown SJ, Denell RE (1999). Molecular and genetic analysis of the *Tribolium Ultrabithorax* ortholog, *Ultrathorax*.. Dev Genes Evol.

[15] Lewis DL, DeCamillis M, Bennett RL (2000). Distinct roles of the homeotic genes Ubx and abd-A in beetle embryonic abdominal appendage development.. Proc Natl Acad Sci U S A.

[16] Zheng Z, Khoo A, Fambrough D, Garza L, Booker R (1999). Homeotic gene expression in the wild-type and a homeotic mutant of the moth Manduca sexta.. Dev Genes Evol.

[17] Stern DL, Emlen DJ (1999). The developmental basis for allometry in insects.. Development.

[18] Mihajlovic M (2005). Utilizing molecular probes to examine diversification of *Oncopeltus fasciatus* and *Acheta domesticus* body plans..

[19] Tomoyasu Y, Wheeler SR, Denell RE (2005). Ultrabithorax is required for membranous wing identity in the beetle Tribolium castaneum.. Nature.

[20] Zhang H, Shinmyo Y, Mito T, Miyawaki K, Sarashina I (2005). Expression patterns of the homeotic genes Scr, Antp, Ubx, and abd-A during embryogenesis of the cricket Gryllus bimaculatus.. Gene Expr Patterns.

[21] Castelli-Gair J, Akam M (1995). How the Hox gene Ultrabithorax specifies two different segments: the significance of spatial and temporal regulation within metameres.. Development.

[22] Peterson MD, Rogers BT, Popadic A, Kaufman TC (1999). The embryonic expression pattern of labial, posterior homeotic complex genes and the teashirt homologue in an apterygote insect.. Dev Genes Evol.

[23] Stern DL (2003). The Hox gene Ultrabithorax modulates the shape and size of the third leg of Drosophila by influencing diverse mechanisms.. Dev Biol.

[24] Averof M, Patel NH (1997). Crustacean appendage evolution associated with changes in hox gene-expression.. Nature.

[25] Damen WG, Hausdorf M, Seyfarth EA, Tautz D (1998). A conserved mode of head segmentation in arthropods revealed by the expression pattern of Hox genes in a spider.. Proc Natl Acad Sci U S A.

[26] Telford MJ, Thomas RH (1998). Expression of homeobox genes shows chelicerate arthropods retain their deutocerebral segment.. Proc Natl Acad Sci U S A.

[27] Abzhanov A, Popadic A, Kaufman TC (1999). Chelicerate Hox genes and the homology of arthropod segments.. Evolution&Development.

[28] Popadic A, Nagy L (2001). Conservation and variation in Ubx expression among chelicerates.. Evol Dev.

[29] Hughes CL, Kaufman TC (2002). Exploring the myriapod body plan: expression patterns of the ten Hox genes in a centipede.. Development.

[30] Garcia-Bellido A (1975). Genetic control of wing disc development in *Drosophila*.. Cell Patterning.

[31] Akam M (1998). Hox genes: from master genes to micromanagers.. Curr Biol.

[32] Brodu V, Elstob PR, Gould AP (2002). abdominal A specifies one cell type in Drosophila by regulating one principal target gene.. Development.

[33] Rozowski M, Akam M (2002). Hox gene control of segment-specific bristle patterns in Drosophila.. Genes Dev.

[34] Lohmann I, McGinnis W (2002). Hox Genes: it's all a matter of context.. Curr Biol.

[35] Ronshaugen M, McGinnis N, McGinnis W (2002). Hox protein mutation and macroevolution of the insect body plan.. Nature.

[36] Galant R, Carroll SB (2002). Evolution of a transcriptional repression domain in an insect Hox protein.. Nature.

[37] Stern DL (1998). A role of Ultrabithorax in morphological differences between Drosophila species.. Nature.

