# Supplementary figures and images for: Ubx Regulates Differential Enlargement and Diversification of Insect Hind Legs
Source: PLoS One. 2007 Sep 12;2(9):e866. doi: 10.1371/journal.pone.0000866 (PMC1959121; doi:10.1371/journal.pone.0000866)

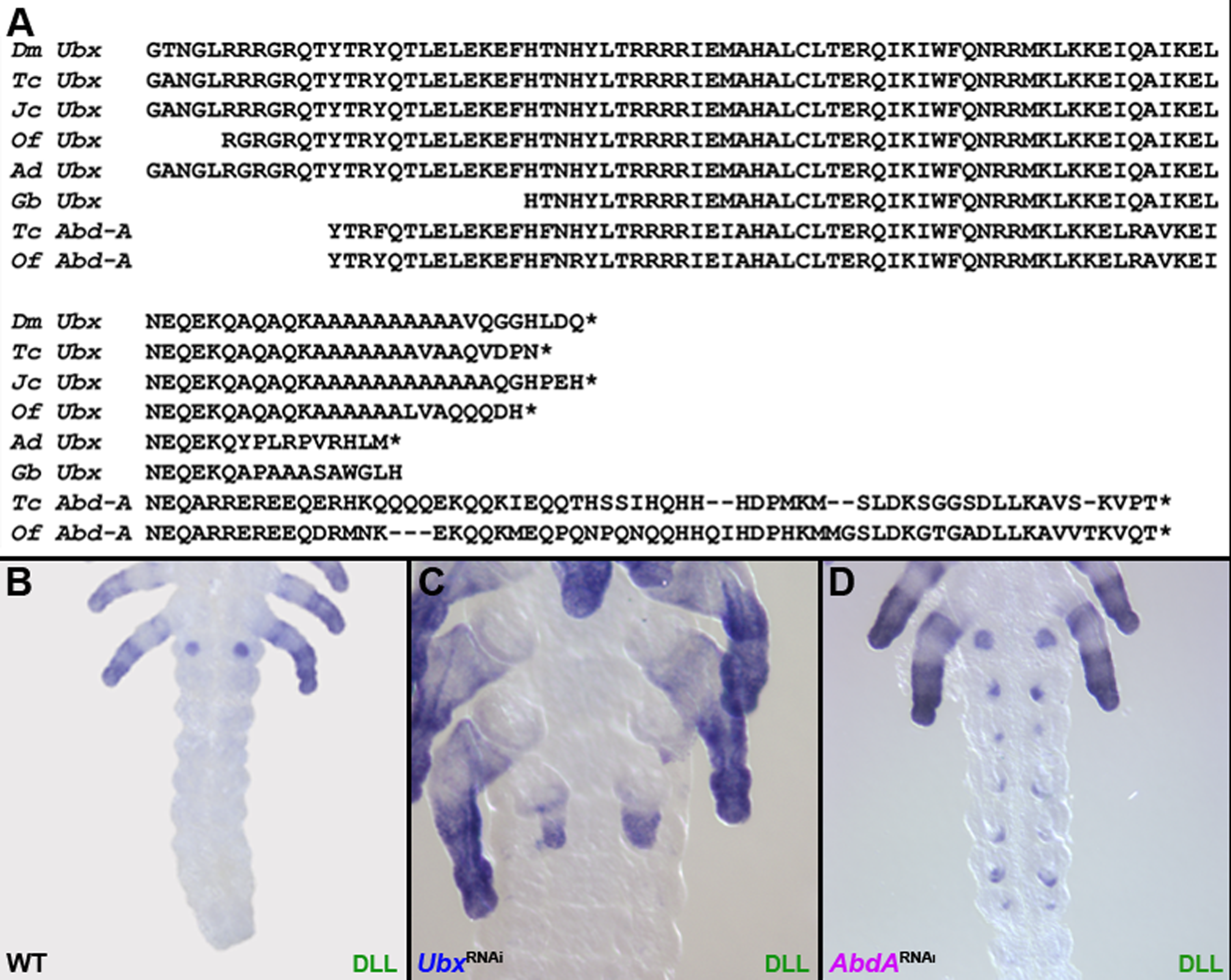

Supplement: Figure S1 — (A) Aligned sequences of cloned Ubx and abd-A cDNA fragments from O. fasciatus and A. domestica. Abbreviations: Dm, Drosophila melanogaster, Tc, Tribolium castaneum, Jc, Junonia coenia, Of, Oncopeltus fasciatus, Ad, Acheta domesticus, and Gb, Gryllus bimaculatus. (B–D) Distal-less (Dll) expression in Oncopeltus wild type, Ubx RNAi, and abd-A RNAi embryos, respectively. In depleted embryos, Dll expression is released in A1 (C), and A2–A8 (D) segments. (4.16 MB TIF) [file pone.0000866.s001.tif]
